# Chronic Expanding Hematoma in the Dorsal Cervicothoracic Region as a Long-Term Complication of Retained Bullet Fragments: Case Report

**DOI:** 10.7759/cureus.858

**Published:** 2016-11-01

**Authors:** Avais Raja, Saima Ahmad, Salman Ehmed, Terri Blume, Emmanuel K Fai, Agha S Khan

**Affiliations:** 1 Department of Orthopaedic Surgery, Shifa College of Medicine, Islamabad, Pakistan; 2 Neurosurgery, Flinders Medical Centre; 3 Emergency Medicine, Kings Edward Medical University; 4 Neurosurgery, University of Maryland Medical Center Midtown Campus

**Keywords:** chronic expanding hematoma, retained bullet fragments

## Abstract

Chronic expanding hematoma is a rare pathology, which has not been previously described as a complication of gunshot injury with retained bullet fragments. Because of the similar characteristics of chronic expanding hematoma to malignancy, it can present a diagnostic challenge for clinicians. Imaging and biopsy evaluation is needed to reach a conclusive diagnosis and implement appropriate treatment. In this case report, we will discuss the development, diagnosis, and management of a chronic superficial cervicothoracic mass in a patient who presented 30 years post-gunshot injury with retained bullet fragments.

## Introduction

Superficial soft tissue masses are common presentations in clinical practice [1]. However, due to the non-specific nature of these lesions, it can be difficult to formulate a conclusive diagnosis [1]. Confusion can arise while assessing superficial masses clinically, particularly when distinguishing chronic expanding hematoma from malignant soft tissue sarcoma [2-3]. Furthermore, due to the rare occurrences of chronic expanding hematoma, which have variable anatomical and morphological characteristics, early and prompt diagnosis is rarely established [2, 4]. Within the literature, chronic expanding hematoma as a complication of a gunshot injury or retained bullet fragments has not been previously reported. In this case report, we will discuss the development and management of a chronic superficial cervicothoracic mass in a patient with a previous gunshot injury and retained bullet fragments. The patient’s informed written consent, for print and electrical distribution of the case report, was obtained. The authors have no conflict of interest in relation to this article. 

## Case presentation

**Figure 1:**
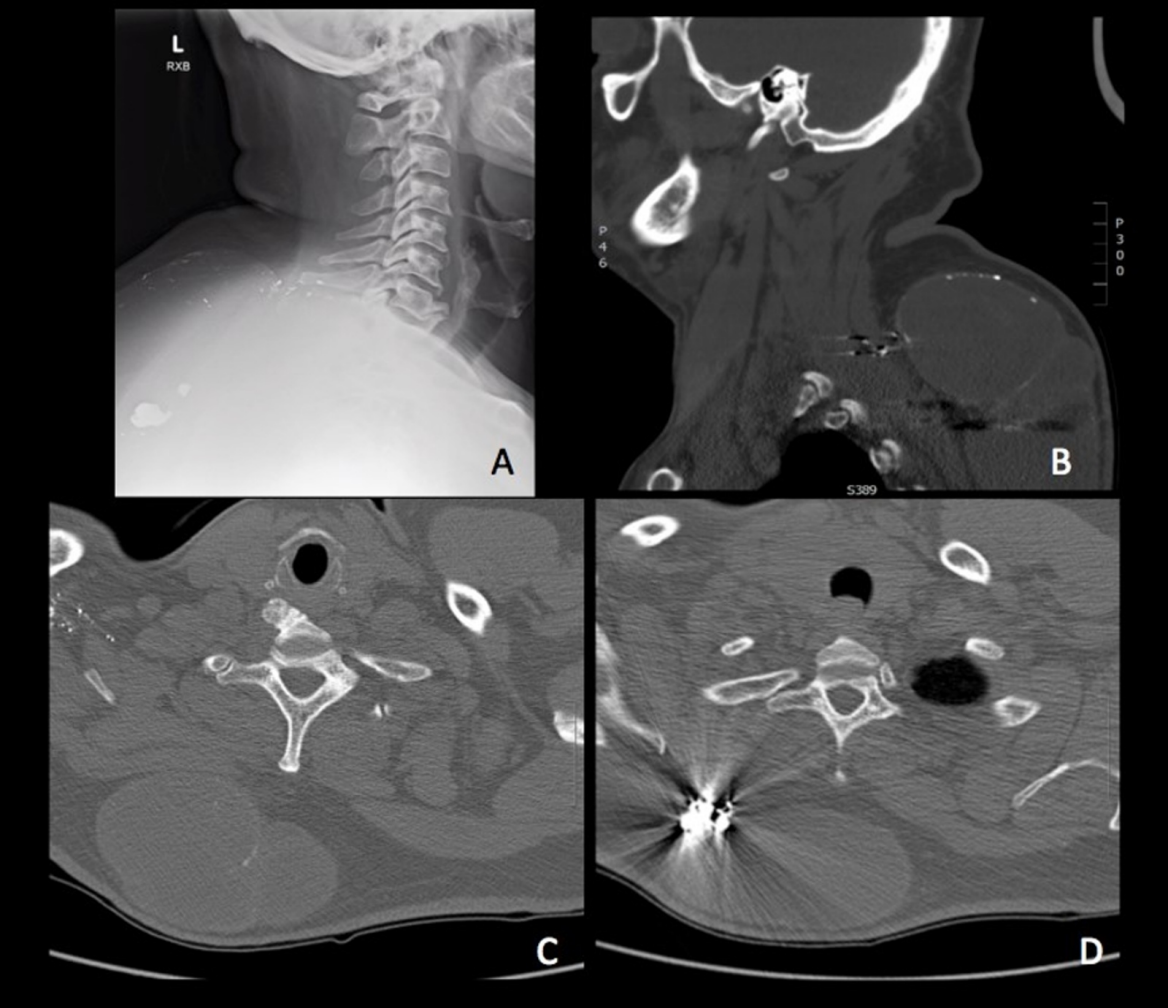
Well-circumscribed, multilobulated subcutaneous lesion with thin septation and ballistic fragments along the margin. Cervical x-ray (A); Non-contrast CT: Sagittal view (B), Transverse views (C and D)

A 52-year-old African American man presented to the neurosurgical clinic with a history of a progressively increasing painful mass in the dorsal cervicothoracic region and difficulty elevating his left arm. The mass was located at the site of a previous gunshot injury with retained bullet fragments sustained 30 years prior. Clinically, the mass was tender to palpation, non-fluctuant, and non-illuminating with no obvious draining lymph nodes. The overlying skin was smooth and non-erythematous. On neurological examination, the patient had difficulty abducting the left arm, most likely due to the compressive nature of the mass. There were no accompanying signs of upper or lower motor neuronal lesions. Computed tomography revealed a well-circumscribed, multilobulated mass within the subcutaneous fat, measuring 10 x 12 x 15 cm and extending from C5 to T5, with ballistic fragments along the peripheral margin [Figure 1]. Informed patient consent was obtained for treatment in this case.

The patient underwent aspirational biopsy of the mass from three different areas. Laboratory analysis of the tissue aspiration consisted of leukocytes and erythrocytes with biochemical values similar to blood. Microbiological and anaerobic cultures were negative. Computed tomography angiogram revealed no significant vascularity or large feeding vessels to the mass. Following these investigations, the patient underwent en bloc resection of the mass from the subcutaneous tissue, thoracodorsal fascia, and base of the neck. Multiple vascular pedicles feeding the mass were identified and resected accordingly.

**Figure 2:**
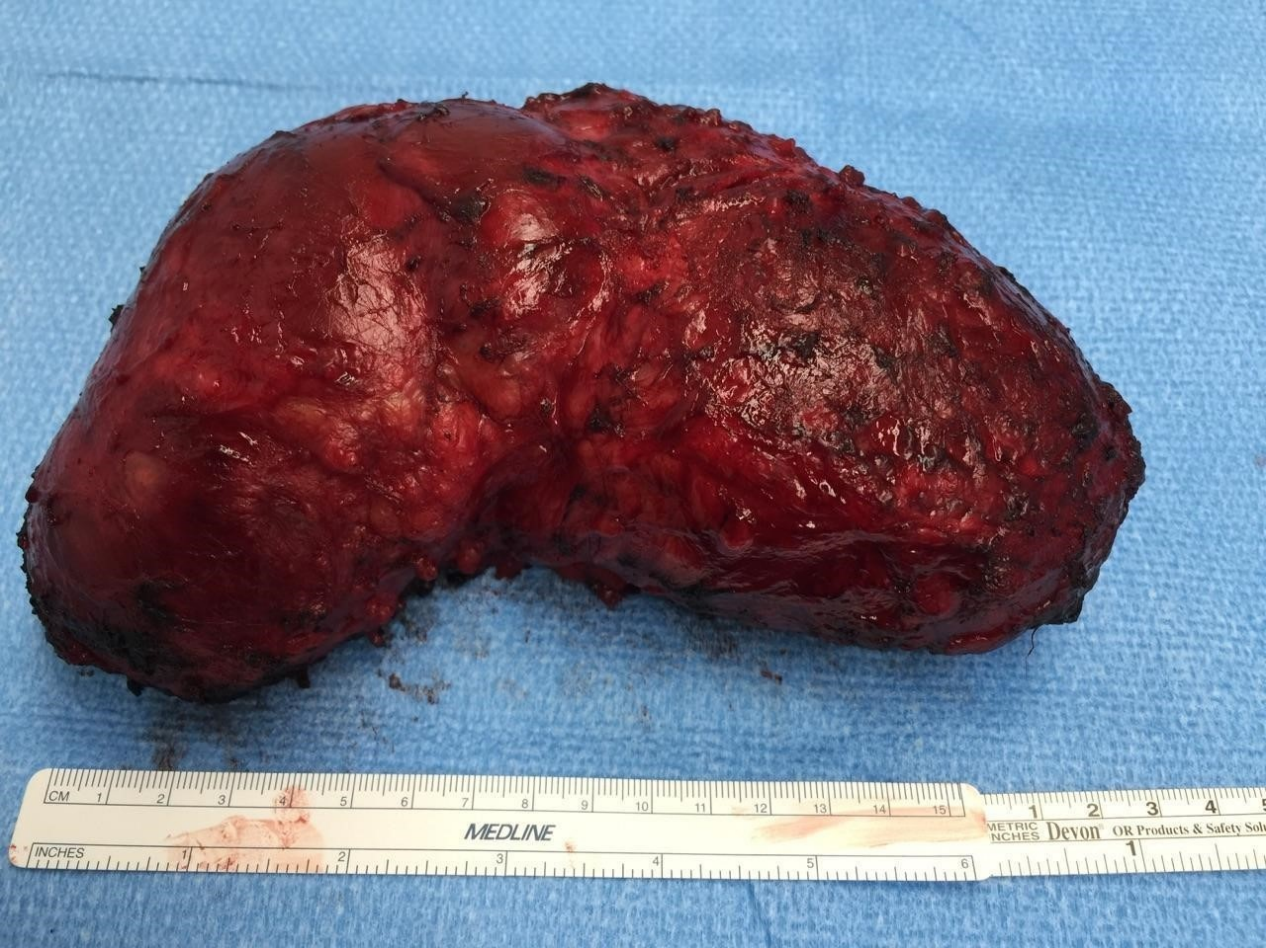
Surgically resected cervicothoracic mass

The resected mass was well-encapsulated, measuring 17.7 x 11.5 x 7.1 cm, with a weight of 810 grams [Figure 2]. The mass was red to yellow in color and partially solid and cystic in nature on further dissection. The solid areas appeared diffusely hemorrhagic while the cystic areas were multiloculated, containing red to brown fluid and friable material. Within the specimen, a piece of metal measuring 1.2 x 0.9 x 0.7 cm resembling a bullet shell was also noted. Histopathologically, the specimen was classified as an organized collection of hematoma associated with foreign giant body cell reaction and calcifications. 

## Discussion

Despite an increase in survival from war injuries and gunshot-related incidents within the general public, the current knowledge of wound ballistics is limited [4-5]. The lethality of bullet injuries is thought to occur in the acute setting, as a consequence of vital organ damage, whilst retained bullet fragments are often considered inert foreign bodies [5]. As such, bullet fragments may be left in situ in cases where they are inaccessible, removal may cause further tissue damage, or the risk of complications due to the retained fragments are low [6].

However, long-term complications of retained bullet fragments have also been described in the literature, including irritative, obstructive, and compressive symptoms near affected organs and neoplastic changes, such as osteosarcoma and myxofibrosarcoma [7-9]. To our knowledge, chronic expanding hematomas have not been reported as complications of gunshot injuries or retained bullet fragments. Database searches using the term ‘chronic expanding hematoma’ on PubMed and Medline, generated only two case reports describing this pathology in the back and no reports secondary to gunshot injury or retained bullet fragments [10].

Chronic expanding hematoma is a rare pathological process that has been defined as an enlarging hematoma that persists for more than a month [7]. It is often associated with previous trauma or surgery but can also occur spontaneously with anticoagulation therapy or in patients with bleeding dyscrasias [8-9]. Within the literature, this tumor-like lesion has been described in a range of anatomical locations with a higher incidence reported in the extremities [8-10]. Furthermore, the size of lesions have also been variable, and clinically latent periods range from months to years [9-10].

The etiology of chronic expanding hematoma remains ambiguous; however, a number of theories have been presented within the literature [3, 8-9]. Displacement of subcutaneous tissue due to trauma or surgery may lead to the formation of a potential space, which fills with blood [9]. A dense fibrous capsule forms around this area and further expansion may occur as a result of the released inflammatory mediators and breakdown products [8]. These substances are thought to impact the coagulation cascade and local vasculature and create a high osmotic pressure gradient, thus, causing further inflammation and bleeding [8]. As these lesions can mimic malignant tumors (in particular, soft tissue sarcoma), they present a diagnostic dilemma for clinicians [2-3].

## Conclusions

This report presented a case of chronic expanding hematoma developing in the dorsal cervicothoracic region 30 years post-gunshot injury with associated retained bullet fragments. Due to the rarity of this pathology and similar characteristics to malignancy, it presented a diagnostic challenge. The treatment of choice is surgical resection of the lesion, which was performed in this patient.
